# Clinical utility of the Montgomery-Åsberg Depression Rating Scale for the detection of depression among bariatric surgery candidates

**DOI:** 10.1186/s12888-016-0823-8

**Published:** 2016-04-30

**Authors:** Leorides Severo Duarte-Guerra, Clarice Gorenstein, Paula Francinelle Paiva-Medeiros, Marco Aurélio Santo, Francisco Lotufo Neto, Yuan-Pang Wang

**Affiliations:** Department and Institute of Psychiatry (LIM-23), University of São Paulo Medical School, São Paulo, Brazil; Department of Pharmacology, Institute of Biomedical Sciences, University of São Paulo, São Paulo, Brazil; Department of Surgery, University of São Paulo Medical School, São Paulo, Brazil; Instituto & Departamento de Psiquiatria, Hospital das Clínicas, Faculdade de Medicina da Universidade de São Paulo, Rua Dr. Ovídio Pires de Campos. no. 785, 05403-010 São Paulo, Brazil

**Keywords:** Obesity, Depression, Psychopathology, Bariatric surgery

## Abstract

**Background:**

Clinical assessment of depression is an important part of pre-surgical assessment among individuals with morbid obesity. However, there is no agreed-upon instrument to identify mood psychopathology in this population. We examined the reliability and criterion validity of the clinician-administered Montgomery-Åsberg Depression Rating Scale (MADRS) and the utility of a short version for bariatric surgery candidates.

**Methods:**

The sample was 374 patients with obesity, consecutively recruited from the waiting list of a bariatric surgery clinic of University Hospital, Brazil: women 80 %, mean BMI 47 kg/m^2^, mean age 43.0 years. The 10-item MADRS was analyzed against the SCID-I. Items that showed small relevance to sample’s characteristics and contribution to data variability were removed to develop the short 5-item version of scale. We calculated the sensitivity and specificity of cutoff points of both versions MADRS, and values were plotted as a receiver operating characteristic curve.

**Results:**

For the 10-item MADRS, the Cronbach’s alpha coefficient was 0.93. When compared against SCID-I, the best cut-off threshold was 13/14, yielding sensitivity of 0.81 and specificity 0.85. Following items were removed: reduced appetite, reduced sleep, concentration difficulties, suicide thought and lassitude. The 5-item version showed an alpha coefficient of 0.94 and a best cut-off threshold of 10/11, yielding sensitivity of 0.81 and specificity 0.87. Similar overall ability to discriminate depression of almost 90 % was found for both 10-item and 5-item MADRS.

**Conclusion:**

The MADRS is a reliable and valid instrument to assess depressive symptoms among treatment-seeking bariatric patients. Systematic application of the abbreviated version of the MADRS can be recommended for enhancing the clinical detection of depression during perioperative period.

**Electronic supplementary material:**

The online version of this article (doi:10.1186/s12888-016-0823-8) contains supplementary material, which is available to authorized users.

## Background

Depression and obesity are onerous non-communicable conditions that frequently coexist [1]. Depressive disorder is one of the most common psychiatric comorbidities among patients with severe obesity (BMI > 40 kg/m^2^), in the pre- and the post-surgical period [2, 3]. Potential bi-directional relationship between obesity and depression is observed and possible causal relationship between these conditions is suggested [4]. Although pre-surgery depression alone has not been shown to be a robust outcome predictor of post-operative weight loss [5], its presence in pre-surgical period can contraindicate bariatric procedure [6]. Therefore, the accurate identification of psychopathology is a crucial step in these patients.

Most of bariatric clinicians acknowledge that depression should be treated and stabilized before surgery, but there are problems in its detection. Non-recognition of depressive symptoms can result in misdiagnosis [7] and unreliable judgment of surgical eligibility [8]. Moreover, many patients tend to present themselves in positive light during psychosomatic evaluations, highlighting somatic symptoms to meet medical expectations and clinical guidelines of pre-surgical assessment. This impression management represents an effort to control or influence the perceptions of healthcare staff in the approval process [6, 9].

The performance of symptomatic scales of depression can be altered when somatic and cognitive symptoms co-occur alongside of depressive symptoms [9], what can also lead to misclassification and mistakes in surgical indication. While overdetection of depression can increase disapprovals to bariatric surgery [6, 9], its underdetection can cause unfavorable outcome among unrecognized depressive patients in post-surgical periods [10]. Thus, the adoption of accurate methods displaying acceptable sensitivity and specificity to identify perioperative psychopathology can reduce the burden of depression among bariatric patients [11].

Some popular instruments for detection of depression were used in this population, e.g., the Hamilton Depression Rating Scale – HAM-D [12, 13], the Beck Depression Inventory - BDI [9, 14–20], the Hospital Anxiety and Depression Scale - HADS [21–23], and the Patient Health Questionnaire - PHQ [20, 24, 25]. Most of tools are self-administered for screening depression and some researchers claim that these scales are conceptually flawed for use in specific settings and patients [9, 13, 26], with several psychometric limitations (e.g., poor reliability or validity) unsuitable to determine bariatric surgery.

Among available self-report scales, the BDI is the most common one reported with the purpose of pre-surgical screening for depression among obese populations [9, 15–20]. However, self-reporting instruments like the BDI yield low level of depressive symptoms and significant proportion of false-positive cases [15], due to patient’s characteristics and response style in bariatric setting during preoperative assessment [9]. The HADS does not include somatic symptoms and is viewed as an easy tool to administer in this population; this instrument has being adopted as indicator of psychopathology in a large prospective Swedish Obese Subjects (SOS) trial [22]. Nevertheless, its applicability can be questioned for discriminating depressive illness, as this tool has presented low sensitivity among breast cancer participants [26].

Structured clinician-administered scales for depression have not been well studied in bariatric population. In comparison to the HAM-D, the Montgomery–Åsberg Depression Rating Scale - MADRS [27] encompasses limited number of somatic symptoms. Moreover, the decision for choosing MADRS instead of HAM-D eventually rest on consensus that a useful instrument should capture adequately the construct of depression among patient samples [11], with empirical evidence of psychometric robustness and cost-effectiveness [28].

Given the need for standardized assessment tools that have validity evidence in bariatric population and clinician-administered scales for depression have not been well studied in this population, we investigated the applicability of the MADRS for a sample of pre-surgical patients in the waiting list of a bariatric clinic. The MADRS has never been applied to preoperative bariatric candidates and incorporates a structured interview embedded in a brief 10-item scale [29]. We aimed to determine the accuracy of the scale (a) to estimate its reliability and validity for assessing depression, and (b) to test whether it is possible to develop a short version of instrument without somatic-cognitive symptoms.

## Methods

This validation study determined the performance of the MADRS for assessing depression among patients seeking bariatric surgery.

### Sampling and recruitment

The participants were recruited from a University-based bariatric center in Brazil and should meet the criteria of class III obesity (BMI ≥ 40 kg/m^2^) or class II (BMI ≥ 35 kg/m^2^) with medical comorbidities. They were ranked in a waiting list in accordance to admission date in the program and clinical severity.

The first 500 eligible patients in the list were consecutively invited by telephone to participate in the study. After brief explanation, 63 patients declined to participate. Additional 63 patients were excluded during the assessment period due to: severe psychiatric illness (*n* = 2), previous bariatric surgery (*n* = 5), mobility difficulty (*n* = 37), and incomplete interviews (*n* = 19). The final sample was comprised of 374 individuals, with a participation rate of 74.8 %.

Table 1 shows the demographic characteristics of participants. Of 374 participants, the majority were women (79.9 %). Regarding marital status, half of them were married/cohabiting (50.8 %), one-quarter was single (25.4 %), the remaining was separated/divorced (16.8 %) or widowed (7.0 %). The mean schooling was 9.6 years of education (standard deviation [SD] 3.5), being 35.6 % with 8 years and 44.9 % with 11 years. The mean age was 43.0 years (SD 11.6) and the mean BMI was 47.0 kg/m^2^ (SD 7.1; range 31.2–92.1).

**Table 1:** Demographic characteristics of the sample (*n* = 374)

Characteristic	*N*	(%)
Sex		
Female	299	79.9
Male	75	20.1
Marital status		
Married/cohabiting	190	50.8
Single	95	25.4
Separated/divorced	63	16.8
Widowed	26	7.0
Education		
8 years	133	35.6
11 years	168	44.9
15 years +	73	19.5
Mean age, years (S.D.)	43.0 (11.6)	
Mean BMI, kg/m^2^ (S.D.)	47.0 (7.1)	

### Instruments

- Montgomery-Åsberg Depression Rating Scale (MADRS) [27] investigates the presence of affective, somatic, cognitive and behavioral symptoms of depression. Ten symptoms are rated on a 0–6 scale, along the possible scores of 0–60. The total score classifies the patients in levels of severity: normal or absent 0–6; mild 7–19; moderate 20–34; and severe 35–60 [30]. We used the Portuguese version of MADRS [31] with the aid of the Structured Interview Guide for the MADRS (SIGMA) [29] to assess the anchor points of each item. The raters were clinical psychologists with experience in bariatric patients, whose scores were calibrated in 3 consensus meetings. Previous psychometric study [32] reported the intraclass correlation coefficient (ICC) of the MADRS as acceptable for unipolar depression, ranging between 0.83 and 0.86. These interviewers were blind to patients’ psychiatric diagnosis and rated independently depressive symptoms from November 2010 to March 2012, totaling 17 months. The duration of the SIGMA ranged from 10 to 40 min, without refusal.
- Structured Clinical Interview for DSM-IV Axis I Disorder (SCID-I) [33] is a standardized semi-structured interview generally accepted as gold standard to make diagnosis of psychiatric disorders [34]. From 2010 to 2012, participants were randomly assigned to be face-to-face assessed by trained psychologists with previous experience in obesity and bariatric surgery, with an inter-rater *kappa* estimated as 0.81 [35]. The duration of the interview ranged from 60 to 90 min for the SCID-I. The psychiatric diagnoses of this sample can be inspected in Additional file 1: Table S1.

### Statistical analysis

Descriptive analysis depicted mean MADRS item endorsement rate, standard deviation (SD) and range. The Pearson’s coefficient of variation (CV), Cronbach’s alpha coefficient (α) and item commonality (*h*^2^) were calculated for each item and total score. Non-relevant symptoms were eliminated in accordance with following rationale: (a) clinic wisdom, (b) item endorsement and (c) commonality values. To inspect the between-variable structure of correlation, the dimensionality of the MADRS was examined through factor analysis [36].

Subsequently, the signal detection analysis was performed to establish the best cut-off point by adopting the diagnosis of SCID-I/DSM-IV major depressive disorder as gold standard. The sensitivity and specificity, the positive and negative predictive values (PPV/NPV) were calculated for all possible thresholds. The Youden’s index (γ) [37] was calculated to summarize the overall misclassification. The Receiver Operating Characteristic (ROC) curve was built by plotting data of sensitivity and false-positive rates. The values of Area Under the Curve (AUC) and their respective 95 % confidence intervals (CI) were calculated.

All analyses were performed using SPSS version 20.0 and the level of significance was set at *p* < 0.05 for two-tailed tests.

## Results

Among 374 participants, 7 % (*N* = 26) met the criteria for major depressive episode and 27.5 % (*N* = 103) lifetime major depression in accordance with SCID-I/DSM-IV criteria. The mean total score of MADRS was 7.73 (SD 11.33, range 0–58, CV 1.47). The highest item endorsement rates (mean score around 1.0) were observed for ‘apparent sadness’, ‘reduced sleep’, ‘reported sadness’ and ‘inner tension’. The lowest endorsement rates (mean < 0.75) were ‘suicidal thoughts’, ‘reduced appetite’, ‘pessimistic thoughts’ and ‘concentration difficulties’. The items with the highest dispersion (CV > 2.0) were ‘suicidal thoughts’, ‘reduced appetite’ and ‘pessimistic thoughts’, indicating sizable response bias of score precision (Table 2). Although women scored higher than men (8.08 vs. 6.33), high data dispersion has cancelled the statistical difference between sexes (*p* = 0.23).

**Table 2:** Mean, standard deviation (SD), coefficient of variation (CV) of the Montgomery-Åsberg Depression Rating Scale (MADRS) for severely obese patients (*N* = 374). Item-total correlation and commonalities

Item	Mean	SD	CV	*α*	*h* ^*2*^
Apparent sadness	1.10	1.70	1.55	0.92	0.79
Reported sadness	0.99	1.72	1.74	0.92	0.77
Inner tension	0.94	1.47	1.56	0.92	0.71
Reduced sleep	1.00	1.63	1.63	0.93	0.41
Reduced appetite	0.48	1.08	2.25	0.94	0.33
Concentration difficulties	0.74	1.39	1.88	0.93	0.62
Lassitude	0.76	1.38	1.82	0.92	0.67
Inability to feel	0.76	1.45	1.91	0.92	0.81
Pessimistic thoughts	0.63	1.35	2.14	0.92	0.74
Suicidal thoughts	0.33	0.99	3.00	0.93	0.49
Total	7.73	11.33	1.47	0.93	0.63^**a**^

CV Pearson’s coefficient of variation = SD/Mean

*α* Cronbach’s alpha coefficient of internal consistency

*h* ^*2*^ Commonality

^a^Total percentage of data explained by unidimensional model of the MADRS

For the 10-item scale, the Cronbach’s alpha coefficient was 0.93, showing adequate homogeneity. Item-total correlation demonstrated that deleting any item would affect slightly the internal consistency of the construct (Cronbach’s alpha range 0.92–0.94). Conversely, the analysis of commonalities indicated that following somatic-cognitive items explained smaller proportion of data variability (*h*^*2*^ < 0.7), in decreasing order: ‘reduced appetite’, ‘reduced sleep’, ‘suicidal thoughts’, ‘concentration difficulties’ and ‘lassitude’.

The sensitivity, specificity, PPV, NPV and best cut-off point for scores of the 10-item and 5-item MADRS are presented in Table 3. For 10-item MADRS, the best trade-off threshold between sensitivity and specificity was 13/14, yielding sensitivity 0.85 and specificity 0.81. The PPV and NPV were 0.70 and 0.91, respectively. The highest Youden’s index was γ = 0.66. Further analysis showed that 23.8 % of participants scored above 13 points with mean score of 24.96 (25.1 % women and 18.7 % men).

**Table 3:** Sensitivity, specificity, positive and negative predictive values (PPV and NPV) and best cut-off point for scores of the 10-item and 5-item MADRS

10-item MADRS											
Cut-off	**5/6**	**6/7**	**7/8**	**8/9**	**9/10**	**10/11**	**11/12**	**12/13**	**13/14** ^**a**^	**14/15**	**15/16**
Sensitivity	0.92	0.92	0.92	0.90	0.87	0.85	0.85	0.85	0.85	0.77	0.69
Specificity	0.64	0.66	0.69	0.70	0.74	0.76	0.77	0.81	0.81	0.83	0.84
PPV	0.52	0.56	0.56	0.57	0.59	0.60	0.62	0.69	0.70	0.72	0.72
NPV	0.95	0.95	0.95	0.94	0.93	0.92	0.92	0.91	0.91	0.86	0.82
γ	0.56	0.58	0.61	0.62	0.62	0.61	0.62	0.66	0.66	0.60	0.53
5-item MADRS											
Cut-off	**5/6**	**6/7**	**7/8**	**8/9**	**9/10**	**10/11** ^**a**^	**11/12**	**12/13**	**13/14**	**14/15**	**15/16**
Sensitivity	0.88	0.88	0.85	0.81	0.81	0.81	0.69	0.65	0.58	0.58	0.46
Specificity	0.75	0.76	0.81	0.85	0.86	0.87	0.88	0.90	0.90	0.90	0.93
PPV	0.57	0.56	0.61	0.70	0.71	0.75	0.74	0.78	0.76	0.76	0.81
NPV	0.71	0.94	0.94	0.91	0.91	0.90	0.85	0.82	0.79	0.79	0.72
γ	0.63	0.64	0.66	0.66	0.67	0.68	0.57	0.55	0.48	0.48	0.47

γ Youden’s index = (sensitivity + specificity) - 1

^a^Maximum trade-off between sensitivity and specificity

Bold-data: MADRS' threshold scores

The factor analysis demonstrated a unidimensional structure of ‘general depression’ for MADRS, explained 63.4 % of data variability and salient factor loadings > 0.4 in all items (not shown). Because ‘reduced appetite’, ‘reduced sleep’ and ‘suicidal thoughts’ showed lower commonalities (*h*^*2*^ < 0.5), and ‘concentration difficulties’ and ‘lassitude’ showed moderate commonalities (*h*^*2*^ < 0.7), these somatic-cognitive items were sequentially removed to develop short versions of scale. While ‘pessimistic thoughts’ presented high CV of 2.14, its contribution to the underlying construct was substantial (*h*^*2*^ = 0.74) and was retained in the final version.

The 5-item version presented Cronbach’s alpha of 0.94 and showed comparable performance with 10-item version, in terms of sensitivity and specificity (Table 3). For the best threshold of 10/11, 19.0 % of the sample scored above 10 points (19.4 % women and 17.3 % men).

**Fig. 1:**
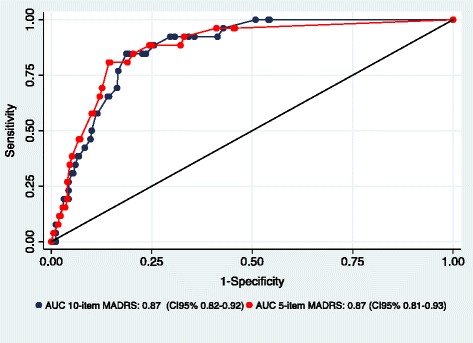
Receiver operating characteristics (ROC) curve and Area Under Curve (AUC) of the 10-item and 5-item MADRS for severely obese patients (*N* = 374)

The sensitivity and false-positive rates (1 - specificity) were used to construct the ROC curve (Fig. 1). The area under the curve (AUC) of 0.87 showed that the discriminative accuracy of scale was satisfactory for both 10-item and 5-item versions. Their respective CI95 % of 0.82–0.92 and 0.81–0.93 indicated substantial overlap between two versions of the scale. In summary, these acceptable results indicated that the MADRS was appropriated for the detection of depression.

## Discussion

Widely adopted in follow-up studies and clinical trials in medical samples it is the first time that the applicability of MADRS was psychometrically investigated among patients with severe obesity. We have shown that the MADRS could be used with good confidence, high sensitivity and specificity in this population. Also, this clinician-administered scale could be customized into a short version, preserving robust psychometric properties. Accurate detection of depression and proper indication of individuals to surgical intervention present a potential impact for public health.

The majority of psychometric studies investigating MADRS confirm its satisfactory reliability, with Cronbach’s alpha close to 0.8 and over [38]. Reliable preoperative evaluations may ensure the reproducibility of pre-operative assessments and improve post-operative outcomes [6, 8].

Where to place the cut-off point to determine the presence of relevant depressive symptoms is critical to the domain of public health. Among clinical studies on MADRS, researchers reported the best threshold ranging from 6 to 21. The large range of cut-off scores indicates the need of validity investigation to ensure its applicability for specific populations. Similar to present study, three studies reported the best cut-off of 13/14 [39] for neurodegenerative patients, one study recommended 6/7 for bipolar patients [40] and another 20/21 for geriatric sample [41].

Concerning the severity level of depression, the mean score of 7.7 could detect cases of “mild depression” [30]. Highly sensitive threshold of 6/7 can be recommended for screening purpose in community studies.

Examining the threshold region between 6 and 9, the scale still has detected cases of depression with substantial sensitivity. However, the specificity of approximately 70 % along with lower PPV (<0.6) was of limited help to identify active cases of depression. While the sensitivity has slightly reduced in the region between 10 and 14, substantial improvement in specificity was observed. The recommended threshold for patients with severe obesity should be 13/14 (mean score around 25), where cases were classified as “moderate depression” with higher specificity. This versatile performance of MADRS can refer cases with different level of depression and settings, for example, in pre-surgical period.

The clinician should be mindful of the disease and sample’s characteristics to interpret the scores of MADRS. The tolerable number of cases of false-positives or false-negatives should be taken into account. Particularly, the low endorsement rate of ‘reduced appetite’ in patients with obesity indicates that this symptom can decrease the total score and the identification of depression in over-eating individuals. Similarly, the inclusion of ‘suicidal ideation’, which is infrequently observed among high BMI individuals [42], can lead to inconsistent assessment. If these symptoms are included in the scale, many depressed patients will go unnoticed and remain false-negative cases, even they present clinical depression.

Clinical characteristics associated with obesity may obscure the pattern of reported symptoms by this medical population [43]. For example, obesity-related sleepiness and fatigue are often described by overweight patients and can be misinterpreted as depressive symptom by respondents. Thus, the items ‘reduced sleep’ and ‘lassitude’ also was removed in the short version.

Considering the sample’s characteristics, most of participants were waiting for years to undertake bariatric surgery. Possibly, these eager patients have inclined to respond to evaluation in order to appear positively appropriate to do so [7]. This illness behavior may have affected the truthfulness of answers to interviews regarding the presence of psychopathology [2]. The supposition that some patients might have exaggerated, disguised or minimized the severity of depressive symptoms is compatible with data dispersion of current investigation. This type of response bias can be minimized with observer scales such as the MADRS and its standardized schedule SIGMA. Possible sex difference affecting the performance of long and short version of the MADRS should be tested in a larger size sample.

Finally, it is advocated that the retention of affective symptoms can effectively identify depressive syndrome with confidence and accuracy with the 5-item version. Somatic-cognitive symptoms of depression should be discarded during preoperative assessment of bariatric patients [39]. The positive acceptance of the short scale by the users, both patients and overloaded clinicians, can increase the cost-effectiveness of assessment for inexpensive implementation in bariatric clinics.

### Limitations

Some limitations of this study should be considered before generalizing the results of this investigation to population with severe obesity. All participants were recruited from a single university hospital, raising reservations about the representativeness of participants. However, large sample size and high participation rate support key findings of our data.

Impression management operates among preoperative bariatric patients and alters the results of symptomatic scales and clinical interviews [2, 6, 8]. These patients have produced imprecise responses (large SD and CV indicators), suggesting that most of widely spread deviations from the central tendency was due to their response style. Although we have adopted rigorous SCID-I interview and SIGMA schedule for assessing depression, the social desirability bias can only be minimized, but not completely ruled out [7]. In this context, higher specificity of the face-to-face MADRS can curb the proneness of false-positive cases observed in self-report scales [9].

Although we have not directly compared competing symptom scales in the same sample, available psychometric indicators have shown that the MADRS outperforms the results of previous studies with the BDI in bariatric patients [9, 15], yielding higher specificity and lower misclassification rate. Detection of 87 % of cases of depression [39] by MADRS seems to have reduced the tendency of bariatric patients to mask their emotional and physical symptoms [44]. Somatic-cognitive complaints may increase the total BDI score, confounding real medical complaints with depressive ones [9] and reducing its clinical utility. In several instances, different methodology of data collection [6] and adaptation of psychometric tools [39] may be crucial to meet some specific features of study population.

## Conclusion

Systematic application of an instrument for assessing depression in bariatric sample must be based on current knowledge of patient’s illness behavior in clinical setting, taking into account its psychometric utility and empirical strength. Though there is no agreed-upon guideline for assessing depression among patients with severe obesity, formal mental health evaluation with reliable methods before surgical procedure is recommended [11]. In line with the literature, our investigation showed that the assessment of depression among patients with severe obesity is slippery and requires great caution, where independent interviewers should disregard misleading somatic-cognitive symptoms of depression during preoperative assessment of bariatric surgery.

The forecast that both obesity and depression are epidemic conditions that will rise exponentially in next decades challenges their proper identification as a medical task of utmost interest. Both 10-item and 5-item version of MADRS are effective tools in pre-operative bariatric evaluation of depression, but the abbreviated scale which has removed somatic-cognitive characteristics of treatment-seeking individuals can redress intrinsic inadequacies of the original scale. Systematic assessment with short version of MADRS to detect depression, coordinated screening of patient's weight and metabolic indicators are pivotal to enhance perioperative evaluations of patients with obesity and improve their treatment outcome.

### Ethics and consent to participate

The Ethics Committee of the University of São Paulo approved the study. All participants have signed an informed consent after the assurance that their participation would not influence the eligibility for surgery. There were no refusals to interviews.

### Consent to publish

Not applicable.

### Availability of data and materials

All data supporting the findings is contained within the article and the supplementary material.

## Additional file

Additional file 1: Table S1. Frequency of current psychiatric diagnosis for severely obese patients, in accordance with Structured Clinical Interview for DSM-IV Axis-I Disorders. Total sample (*N* = 374) and by sex. (DOC 44 kb)

## Abbreviations

AUC: area under the curve
BDI: Beck Depression Inventory
BMI: body mass index
CI: confidence interval
CV: coefficient of variation
DSM-IV: Diagnostic and Statistical Manual of mental disorders, fourth edition
*h*^2^: commonality
HADS: hospital anxiety and depression scale
HAM-D: Hamilton Depression Rating Scale
ICC: intraclass correlation coefficient
MADRS: Montgomery-Åsberg Depression Rating Scale
NPV: negative predictive value
PHQ: Patient Health Questionnaire
PPV: positive predictive value
ROC: receiver operating characteristics
SCID-I: Structured Clinical Interview for DSM-IV Axis I Disorders
SD: standard deviation
SIGMA: Structured Interview Guide for the MADRS
SOS: Swedish Obese Subjects Trial
SPSS: Statistical Package for the Social Sciences
γ: Youden’s Index
