# Supplementary material for: Clinical utility of the Montgomery-Åsberg Depression Rating Scale for the detection of depression among bariatric surgery candidates
Source: BMC Psychiatry. 2016 Apr 30;16:119. doi: 10.1186/s12888-016-0823-8 (PMC4852448; doi:10.1186/s12888-016-0823-8)
Supplement: Additional file 1: Table S1. — Frequency of current psychiatric diagnosis for severely obese patients, in accordance with Structured Clinical Interview for DSM-IV Axis-I Disorders. Total sample (N = 374) and by sex. (DOC 44 kb) [file 12888_2016_823_MOESM1_ESM.doc]

**Supplementary Table S1**: Frequency of current psychiatric diagnosis for severely obese patients, in accordance with Structured Clinical Interview for DSM-IV Axis-I Disorders. Total sample (*N* = 374) and by sex.

| **Psychiatric disorder All subjects** | **Total**  **(*N* = 374)** | **Women**  **(*N* = 299)** | **Men**  **(*N* = 75)** |
| --- | --- | --- | --- |
|  | ***N* (%)** | ***N* (%)** | ***N* (%)** |
| **Axis-I Psychiatric Disorder** | **219 (58.6)** | **174 (58.2)** | **45 (60.0)** |
| **Bipolar Disorder** | **51 (13.6)** | **40 (13.4)** | **11 (14.7)** |
| Bipolar Disorder II | 28 (7.5) | 21 (7.0) | 7 (9.3) |
| Bipolar Disorder I | 6 (1.6) | 5 (1.7) | 1 (1.3) |
| Bipolar No Other Specification (NOS) | 17 (4.5) | 14 (4.7) | 3 (4.0) |
| **Depressive Disorder** | **30 (8.0)** | **26 (8.7)** | **4 (5.3)** |
| Major Depressive Disorder | 26 (7.0) | 22 (7.4) | 4 (5.3) |
| Dysthymia | 4 (1.1) | 4 (1.3) | - |
| **Anxiety Disorder** | **179 (47.9)** | **144 (48.2)** | **35 (46.7)** |
| Generalized Anxiety Disorder (GAD) | 93 (24.9) | 78 (26.1) | 15 (20.0) |
| Specific Phobia | 67 (17.9) | 55 (18.4) | 12 (16.0) |
| Social Phobia | 35 (9.4) | 27 (9.0) | 8 (10.7) |
| Agoraphobia | 22 (5.9) | 21 (7.0) | 1 (1.3) |
| Anxiety No Other Specification (NOS) | 19 (5.1) | 15 (5.0) | 4 (5.3) |
| Obsessive-Compulsive Disorder (OCD) | 13 (3.5) | 12 (4.0) | 1 (1.3) |
| Post-Traumatic Stress Disorder (PTSD) | 10 (2.7) | 8 (2.7) | 2 (2.7) |
| Panic Disorder | 9 (2.4) | 8 (2.7) | 1 (1.3) |
| **Substance Use Disorder** | **9 (2.4)** | **5 (1.7)** | **4 (5.3)** |
| Alcohol Use Disorder | 9 (2.4) | 5 (1.7) | 4 (5.3) |
| Drug Use Disorder | 1 (0.3) | 1 (0.3) | - |
| **Eating Disorder** | **75 (20.1)** | **60 (20.1)** | **15 (20.0)** |
| Binge Eating Disorder (BED) | 62 (16.6) | 47 (15.7) | 15 (20.0) |
| Bulimia Nervosa | 13 (3.5) | 13 (4.3) | - |
